# Monitoring the compliance of the academic enterprise with the Fair Labor Standards Act

**DOI:** 10.12688/f1000research.10086.2

**Published:** 2017-09-28

**Authors:** Adriana Bankston, Gary S. McDowell

**Affiliations:** 1 The Future of Research, Inc., 848 Brockton Avenue, Abington, MA, USA; 2 Manylabs, 1086 Folsom Street, San Francisco, CA, USA

**Keywords:** FLSA ruling, postdoctoral salary, research enterprise, science policy, biomedical research, research funding, academic labor, institutional policy

## Abstract

**Background**: On December 1, 2016, the Fair Labor Standards Act (FLSA) was due to be updated by the U.S. Department of Labor. Key changes included an increase in the salary threshold for exemption from overtime for working more than 40 hours per week, and indexing the salary level so that it is updated automatically every 3 years. This was predicted to have a profound effect on academe as postdoctoral researchers were mostly paid at a salary below the new threshold. On November 22, 2016, an injunction was granted nationwide, delaying implementation of the updates, which were finally struck down entirely on August 31, 2017. Here we review the key changes to the FLSA, how they came about, and how the postdoctoral population was affected.

**Methods**: We describe recent data collection efforts to uncover what institutions with postdocs were doing to comply with the FLSA.

**Results**: Our data showed that 57% of institutions checked (containing 41% of the estimated postdoctoral workforce in science, engineering and health) had not decided or had no public decision available one month prior to implementation, and only 35.5% of institutions were planning to raise salaries to the new minimum. After the injunction, a number of institutions and the NIH continued with their plans to raise salaries. Overall, despite the removal of a federal mandate, approximately 60% of postdocs are at institutions whose policy is to raise salaries.

**Conclusions**: Our data show uncertainty in postdoctoral salaries in the U.S. prior to implementation of the FLSA ruling. In addition, while some institutions did suspend plans to raise postdoctoral salaries after the injunction, many continued with the raise. The implementation of postdoctoral salary raises may be inconsistent, however, as the legal minimum is still $23,660.

## Background

### Defining the Fair Labor Standards Act (FLSA)

The Fair Labor Standards Act (FLSA) establishes standards such as minimum wage and overtime pay for employees in both the public and private sectors in the United States. Through the FLSA a minimum wage and overtime pay (for working more than 40 hours per week) at 1.5 times the employee's regular rate are guaranteed ( United States Department of Labor, 2016a).

On December 1, 2016, the FLSA was due to be updated by the U.S. Department of Labor (DOL). One key change proposed was an increase in the annual salary threshold for exemption from overtime pay from the 2004 level of $23,660 to $47,476. The other key change was indexing the salary level so that it would be updated automatically every 3 years pegged to the 40th percentile of full-time salaried workers in the lowest-wage Census region. This means that the overtime threshold will be $51,168 in 2020. We first describe the timeline of how these updates were decided, and how they were due to affect the postdoctoral researcher population. We will then describe how a court injunction, and the eventual demise of the updates, affected postdoctoral salary policies in the U.S.

### Updating the FLSA

On March 13, 2014, a memorandum was issued by the White House from U.S. President Barack Obama to Secretary of Labor Thomas Perez, instructing the Department of Labor to investigate updating and modernizing current overtime regulations:

“ *I hereby direct you to propose revisions to modernize and streamline the existing overtime regulations. In doing so, you shall consider how the regulations could be revised to update existing protections consistent with the intent of the Act; address the changing nature of the workplace; and simplify the regulations to make them easier for both workers and businesses to understand and apply (* Obama, 2014 *).”*

On July 6, 2015, the Department of Labor issued a “Notice of Proposed Rulemaking,” soliciting feedback by September 4, 2015. The notice proposed increasing the current exemption salary of $23,660, set in 2004, to $50,440 in 2016, with automatic updates the level every 3 years ( United States Department of Labor, 2016b; Obama, 2015).

On May 18, 2016, the Secretary of Labor, Thomas Perez, gave notice of the final decision on the updates to overtime regulations in the FLSA. The exemption salary would be set at $47,476 (lower than the $50,440 originally proposed) with updates every 3 years determined by future wage growth ( United States Department of Labor, 2016f). The date for implementation was set as December 1, 2016. Therefore, 2 years after the first indication of a change to overtime regulations, and just under a year from the indication of what those changes were likely to be, an additional 6 months allowance was made to prepare for compliance with the new rule.

Efforts to delay implementation of the new rule included H.R.6094, the Regulatory Relief for Small Businesses, Schools, and Nonprofits Act, which passed the U.S. House of Representatives on September 28, 2016 by 246 votes to 177. It was then passed to the Senate on September 29, 2016 ( Congress of the United States of America, 2016) where it awaits action. An emergency motion for preliminary injunction was also filed by 21 States (Nevada; Texas; Alabama; Arizona; Arkansas; Georgia; Indiana; Kansas; Louisiana; Nebraska; Ohio; Oklahoma; South Carolina; Utah; Wisconsin; Kentucky; Iowa; Maine; New Mexico; Mississippi; Michigan) on October 12, 2016 ( Anon, 2016).

### Court injunction and eventual demise of the updates

On November 16, 2016, a preliminary injunction hearing took place to delay implementation of the FLSA ruling. At that point, if no delay were to be imposed, employers needed to comply with the FLSA on December 1, 2016. However, the injunction was granted nationwide on November 22, 2016 by a federal judge. The updates were finally ruled invalid on August 31, 2017 ( Anon, 2017).

### The debate over the FLSA changes and their effect on higher education

The changes to the FLSA proposed on July 6, 2015 stood to make a large impact on higher education. The large rise in the salary threshold for exemption had the potential to affect a wide range of workers in academe. As stated by the College and University Professional Association for Human Resources (CUPA-HR), affected employees could include: “librarians; advisers; counselors; residence hall managers; admissions counselors; financial aid counselors’ student activities officers; human resources professionals and trainers; accountants; head cashiers; textbook managers; ticket managers; alumni relations; fundraising professionals; head of mail services; farm managers; information technology professionals; research and clinical professionals (including many with advanced degrees and those engaged in advanced training such as postdocs); managers in food service, security and building and grounds. Many of these jobs have always been and are well suited to exempt status ( CUPA-HR, 2015a).”

A concerted effort was therefore made to reduce the potential impact of FLSA changes on higher education. A letter to the Department of Labor was coordinated by CUPA-HR on behalf of 18 higher education organizations ( CUPA-HR, 2015a; CUPA-HR, 2015b). Key recommendations made in the letter were: 1) the Department of Labor providing a longer time to adjust to the changes; 2) proposed lower salary level options of: $29,172, $30,004 or $40,352; and 3) rephrasing language to specifically exempt postdocs based on their “trainee” status in a similar manner to medical residents.

Similarly, the Association of American Medical Colleges (AAMC) submitted a letter supporting this position and adding:

“ *Any increase in the salary threshold for exemption should be graduated and incremental. AAMC recommends an initial threshold that does not exceed the National Institutes of Health (NIH) guidelines for postdoctoral stipends, currently set at $42,840 for new trainees in [fiscal year] 2015. In addition, postdoctoral scientists should be considered salaried, FLSA-exempt “learned professionals,” similar to medical residents* ( AAMC, 2015) *.*”

On the other hand, in addition to postdocs themselves commenting on the ruling ( Wexler, 2016), on May 10, 2016 four unions representing postdocs or higher education employees (American Federation of State, County and Municipal Employees; Service Employees International Union; the United Auto Workers and the National Education Association) met with the Department of Labor to argue against institutional calls for exemption for postdoctoral researchers ( Penn, 2016).

Attempts to exempt postdocs and to push for an exemption threshold below current postdoctoral salary levels were unsuccessful. With the announcement of updates to the FLSA, there was a simultaneous announcement that postdocs would not be exempt and would in fact be targeted by the ruling, discussed in the article co-authored by Director of the NIH Francis Collins and the Secretary of Labor Thomas Perez, “Fair Pay for Postdocs: Why We Support New Federal Overtime Rules ( Collins & Perez, 2016).”

Employees must meet a series of tests in order to be exempt from overtime payments. First, they must be paid on a salary basis and not an hourly basis, by the “salary basis test.” Second, their salary must meet the minimum salary threshold of $913 per week or $47,476 annually, by the “salary level test” (which does not apply to doctors, lawyers or teachers). Finally, the employee’s primary job duty must pass the “standard duties test”. The duties test is either an executive exemption (e.g. managing a department), an administrative exemption (e.g. being in a primarily clerical role), or a professional exemption, such as that of a postdoc. Unless all 3 tests are passed, the employee is eligible for overtime payment. For example, a first year postdoc in 2015 earning a salary of $43,692 would pass the salary basis test, would fail the salary level test and pass the standard duties test. Hence the focus placed on the overtime pay threshold.

The Department of Labor issued a summary of the impact that updates to the FLSA have on higher education ( United States Department of Labor, 2016e) and guidance for higher education on compliance with the FLSA ruling ( United States Department of Labor, 2016c). Limits to the impact of this ruling include exemptions for those who are in primarily teaching roles (such as adjunct faculty) and students (including undergraduate and graduate students) earning degrees. However, technical staff who are primarily carrying out benchwork and not clerical work were likely affected by the new ruling.

### The effect of FLSA updates on postdoctoral researchers

From this point, we will focus particularly on postdoctoral researchers in Science, Engineering and Health, as this population has been the focus of our data collection efforts. However all postdocs (in Science, Technology, Engineering and Math (STEM) disciplines, as well as in humanities and social sciences) primarily engaged in research (and not teaching) at an U.S. institution regardless of visa status and salary source are affected by this ruling as follows:

“ *Postdoctoral fellows are employees who conduct research at a higher education institution after the completion of their doctoral studies. Postdoctoral fellows are not considered students because they are not working towards a degree...Postdoctoral fellows often meet the duties test for the “learned professional” exemption but must also satisfy the salary basis and salary level tests to qualify for this exemption.”* ( United States Department of Labor, 2016c)

***Raising postdoctoral salaries in the U.S. under the FLSA.*** Recommendations have been made to raise postdoctoral salaries across a wide swathe of academe, as summarized by Pickett *et al.* ( Pickett *et al.*, 2015). The American Academy of Arts and Sciences ( American Academy of Arts and Sciences, 2014), the National Academies ( Committee to Review the State of Postdoctoral Experience in Scientists and Engineers *et al.*, 2014), senior biomedical researchers ( Alberts *et al.*, 2014), junior scientists ( McDowell *et al.*, 2014), organizations representing postdocs ( National Postdoctoral Association, 2016) and advisory groups to the NIH ( Biomedical Research Workforce Working Group, 2012) have all recommended increases to postdoctoral salaries in the years prior to the FLSA update, often to the level of at least $50,000, which is higher than the proposed level of $47,476 for overtime exemption.

The Department of Labor issued the following statement in its guidance to higher education about current postdoctoral salaries:

“ *Under the 2016 National Institutes of Health (NIH) salary guidelines for postdoctoral research fellows, some fellows earn more than the revised salary level. Other postdoctoral research fellows earn less, although it is the Department’s understanding that many postdoctoral research fellow salaries are close to the new salary level, and that any differences are not more than a few thousand dollars a year* ( United States Department of Labor, 2016c).”

**Figure 1.**
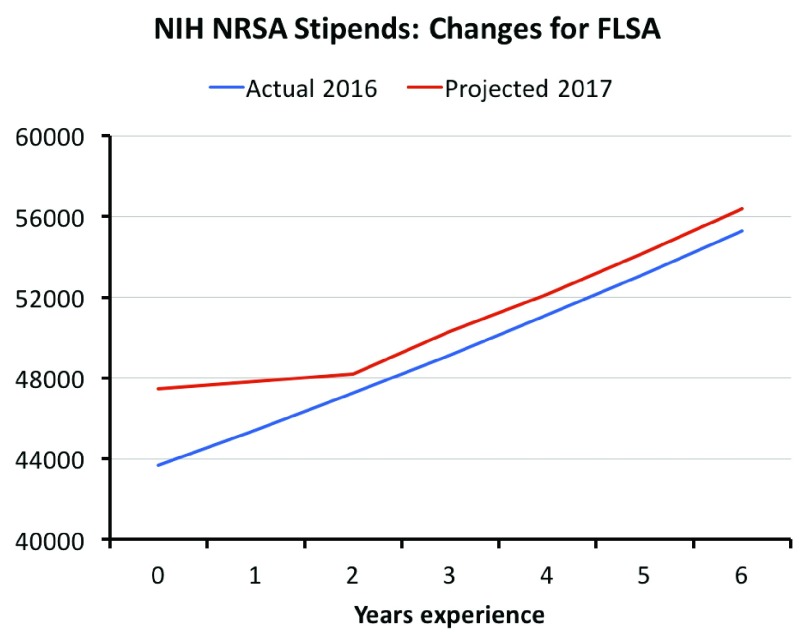
NRSA stipend level changes with FLSA as of December 1st 2016. The 2016 salary for postdocs on NRSA grants (blue) in 2016 increases from a minimum of $43,692 at a linear rate with increasing years of postdoctoral experience. The 2017 levels (red) will raise the minimum salary in 2017 to $47,484, which will remain relatively constant until 2 years experience and increase linearly thereafter (data source: NIH NOT-OD-16-134, ( National Institutes of Health, 2016)).

There is an assumption that postdoctoral salaries are, on average, around $45,000 per year for a full-time postdoc ( Collins & Perez, 2016) and that most institutions follow the NIH National Research Service Award (NRSA) stipend levels. In theory, therefore, the salary changes expected for postdocs in many cases should approximate the changes in the new NRSA levels ( National Institutes of Health, 2016) as shown in Figure 1.

The minimum annual salary should therefore rise from $43,692 for Year 0 postdocs to $47,484, which constitutes an increase of 8.7%. Thus, postdocs with more than 3 years of postdoctoral experience are, in theory, already exempt from this rule. However, it is difficult to gauge exactly how postdoctoral salaries are changing across the U.S. Transparent salary information for postdoctoral positions is very hard to find and the administration of postdocs ( Callier, 2016; McDowell, 2016b; Schaller & McDowell, 2016) means that many of them may be on lower salaries than expected. It is not currently possible for all institutions in the United States to identify and obtain information on the salaries of all of their postdocs with certainty. Therefore we cannot tell whether all postdocs at Year 3 and above are actually currently paid according to the NRSA scale, and so cannot determine how this group is affected.

Another problem with the NRSA assumption is that not all institutions peg their salaries to NRSA levels even for Year 0. In 2014, 89% of institutions had a minimum salary policy, where 51% of those institutions set their postdoctoral salary scales to the NRSA scale, but ~30% set their minima lower and 7% did not enforce these policies (data in Figure 21, ( Ferguson *et al.*, 2014); for a visual description of this see also ( McDowell, 2016)). It is also unclear how many postdocs are employed at each of these institutions (populations potentially from 1 to 5761, according to 2014 data from the National Science Foundation (NSF, ( National Science Foundation, n.d.)). However, we have recently analyzed data from the NSF’s Survey of Graduate Students and Postdoctorates in Science and Engineering and found a wide range of errors in reporting the data year-to-year ( Pickett *et al.*, 2017) and therefore the number of U.S. postdocs at different points on this salary range is also unclear. If 11% of U.S. institutions haven’t set a minimum salary level, it is therefore possible (and legal) that there may be full-time postdocs currently earning as little as $23,660, and these salaries would need to double if they were to meet the FLSA exemption criteria.

International postdocs were also due to be affected by the FLSA overtime rule change. International postdocs, particularly those on temporary visas, are anecdotally supposed to receive lower salaries than U.S. citizens and permanent residents. In a similar manner to the requirements for fellows (see Discussion), nationality is not a condition under the FLSA, but again, it is the nature of the work undertaken which matters; and so international postdocs also came under the ruling.

***Implementing the salary change.*** Institutions had the choice to either increase the minimum salary for postdocs to $47,476, or to classify postdocs as hourly workers.

The first option was difficult because there was no extra money for this ruling, and PIs may have had to pay postdocs from research grants such as R01 grants. Salaries of postdocs on training grants/fellowships (NRSA, HHMI and possibly NSF) were to be increased ( National Institutes of Health, 2016; National Science Foundation, 2016).

The second option, however, may have cost even more. It would involve implementing a system for keeping track of the hours that postdocs spend in the lab. Many postdocs work, and are expected to work, in excess of a standard 40-hour week. One calculation posits that a postdoc earning the NRSA minimum of $43,692 working 50 hours a week under the new rule would have an effective increase in salary to $60,076 ( Polka, 2016).

## Methods

### What were institutions planning to do under the FLSA?

In an effort to make salary information as transparent as possible, we gathered information at the “FLSA and postdocs” resource on the Future of Research website ( http://futureofresearch.org/flsa-and-postdocs/, ( Future of Research, 2016b)). This data-gathering involved checking university websites and contacting HR departments at institutions for information on complying with the FLSA ruling for postdocs. We made it clear that this information was to be made publicly available, using as our guide data from the 2014 NSF Survey of Graduate Students and Postdoctorates in Science and Engineering ( National Science Foundation, n.d.) using the total number of Science, Engineering and Health postdocs as our postdoc population (as all postdocs are affected) to approximate the number of postdocs at each institution, errors in reporting notwithstanding ( Pickett *et al.*, 2017). We were in a position, with one month prior to implementation, to describe the landscape of publicly available information on changes to the administration of postdocs in compliance with the FLSA.

### Council on Governmental Relations Survey - August 2016

The only other publicly available data source was a survey by the Council on Governmental Relations conducted of their membership in August ( Council on Governmental Relations, 2016). Of 190 member institutions, 109 responded, 68 of which had medical schools. Out of these, 79 were public institutions and 30 were private.

In August 2016, 63% of institutions claimed to have made a decision, 19% said the decision would be made in September, and 15% said their decision would be made in October. Therefore 97% surveyed by now, with a month before implementation, should have made a decision. Based on the decisions institutions made or were leaning towards, the survey reported 75% of institutions would raise salaries, and 25% would allow the tracking of hours, 55% of which would leave the decision up to the individual PI. Also, 96 institutions have reported on salary levels with ⅔ reporting at least 50% of salaries, and ¼ reporting at least 75% of salaries were below the new threshold, and 2% reported that 90% of salaries exceeded the new threshold.

### Data collection post-injunction

When the preliminary injunction was granted on November 22, 2016, we began to track how institutions chose to respond to the injunction via checking the university websites or by contacting the HR departments of the same 340 institutions from the 2014 NSF Survey of Graduate Students and Postdoctorates in Science and Engineering ( Pickett *et al.*, 2017; National Science Foundation, n.d.). The data are collected at the Future of Research online resource ( http://futureofresearch.org/flsa-and-postdocs/) under the tab “How institutional plans have/have not changed since the injunction”.

## Results

### One Month Before Implementation

In a blogpost for Addgene, we reported data that had been collected so far ( McDowell, 2016a). In this analysis, we looked at both the percentage of the postdoctoral workforce at institutions implementing various plans for the FLSA, as well as provided data on the percentage of institutions implementing various FLSA plans, as of October 21, 2016.

Repeating this analysis a month before compliance was required, we were able to discuss data for institutions that we had checked or contacted covering 97.5% of the estimated postdoctoral workforce, or every U.S. higher education institution listed in the NSF dataset with > 35 postdocs in 2014 ( Dataset 1).

**Figure 2.**
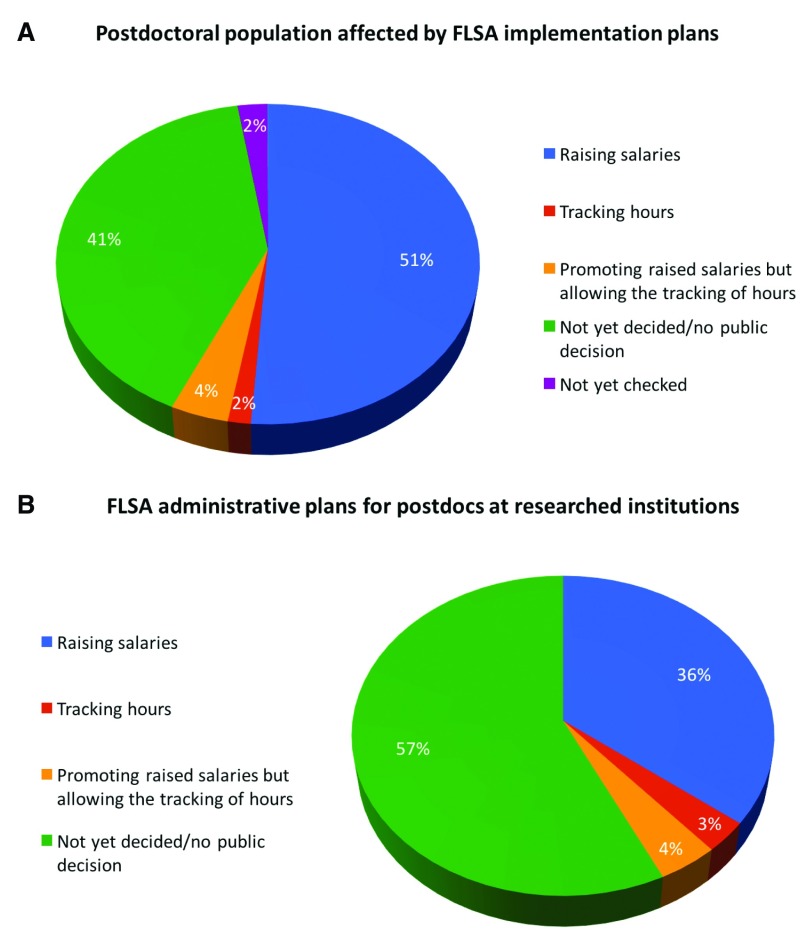
The predicted effect of FLSA updates and institutional decisions on the Science, Engineering and Health postdoctoral workforce as of November 1st 2016. Pie charts show the percentage of the postdoctoral population at institutions implementing various plans for FLSA ( **A**) and the percentage of institutions implementing various plans for FLSA (includes institutions so far researched for the FLSA and postdocs resource) ( **B**).

Out of these, 51% of the estimated postdoctoral workforce came from institutions that had stated they were raising salaries, 1.5% from institutions focused on tracking hours, and 4% from institutions allowing the tracking of hours while promoting (but not mandating) salary raising. However, still 41% of postdocs came from institutions that had either reported to us that they had not decided, had no information available, and/or had not yet responded to a request for information ( Figure 2A).

One month away from December 1, 2016, we had checked 56% of institutions, and of those checked, 35.5% were planning to raise salaries, and 57% had no public decision yet available ( Figure 2B).

### A case in point: Boston postdocs

To illustrate the point of what postdocs knew at this point in time, with one month prior to implementation, we took Boston postdocs as an example. The Boston Postdoctoral Association had been taking an active role in gathering institutional information and preparing resources on the FLSA for its members ( Boston Postdoctoral Association, 2016). We have listed Boston institutions along with the numbers of postdocs from the NSF dataset we used for our analysis (where known; “Harvard” and “MIT” are each listed as a single institution) and current estimates of 9,000 postdocs in Boston ( Table 1).

**Table 1.** Postdoctoral salary status and the number of postdoctoral researchers at various universities in Boston. *Unknown number out of 5,761 at “Harvard” and **Unknown number out of 1,516 at “MIT”.

University	Postdoc salary status	Number of postdocs
Boston Children’s Hospital	Raising salary	Unknown*
Boston University & Boston University Medical Campus	Raising salary	444
Brandeis University	Raising salary	102
Brigham and Women’s Hospital	Unknown	Unknown *
Broad Institute	Unknown	Unknown *
Dana-Farber Cancer Institute	Salary already $50,000	Unknown*
Harvard Medical School & Harvard School of Dental Medicine	Decision not yet made/available	Unknown *
Harvard T.H. Chan School of Public Health	Raising salary	Unknown *
Harvard University	Raising salary	Unknown *
Joslin Diabetes Center	Unknown	Unknown *
Massachusetts Eye and Ear Infirmary	Unknown	Unknown *
Massachusetts General Hospital	Unknown	Unknown *
Massachusetts Institute of Technology	Unknown except for Department of Brain and Cognitive Sciences, which has a salary already of $51,120	Unknown **
Tufts University	Raising salary	194
Whitehead Institute for Biomedical Research	Salary already $50,127 (in 2012)	Unknown **

Out of 9,000 postdocs in Boston, and estimating the distribution of postdocs at Harvard and MIT institutions, we estimate that half, and perhaps as many as two thirds, of the postdocs in Boston - a very well-organized group of postdocs already gathering information - were not aware of what their status would be in a month, either because they had not been told, or their institution had not yet made a decision.

### Changes reported post-injunction

**Figure 3.**
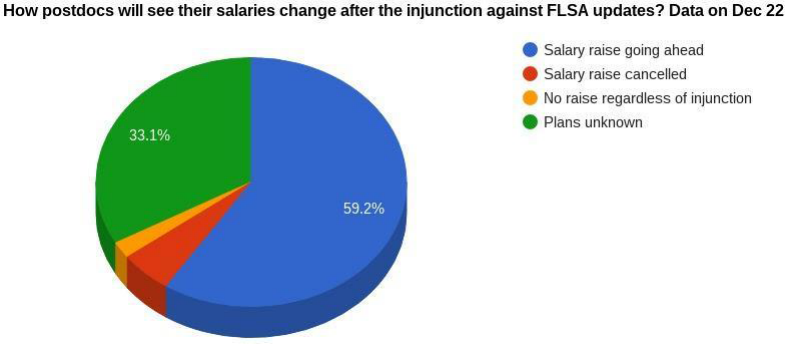
The effect of FLSA updates and institutional decisions on the Science, Engineering and Health postdoctoral workforce after an injunction against the updates was granted (data as of December 22nd 2016). Pie chart shows the percentage of the postdoctoral population at institutions implementing various plans for salary policies after an injunction against the FLSA updates was granted.

Repeating the data gathering process after the injunction, at which point we also received much more input from institutions from which we previously had not received any data, we found that on December 22, 2016, at exactly one month following the injunction, 59.2% of postdocs were still expected to receive salary raises ( Figure 3). This information is documented on the Resource page ( http://futureofresearch.org/flsa-and-postdocs/, ( Future of Research, 2016b)) under the tab, “How institutional plans have/have not changed since the injunction”. We have summarized information to the best of our knowledge in Dataset 1, which lists whether or not we had determined that a FLSA-compliant policy was in place before the injunction (raising salaries and/or tracking hours), and whether institutions raised all salaries after the injunction, using postdoctoral population data from the NSF’s 2014 GSS data tables ( National Science Foundation, n.d.). An updated version of this table will be kept at our website and we can be contacted for corrections and updates ( http://futureofresearch.org/flsa-and-postdocs/, ( Future of Research, 2016b)).

Status of postdoc policies prior to and following the injunction against updates to the FLSA The dataset is a compilation of information obtained from checking HR websites or contacting HR departments at various universities listed in the 2014 NSF Survey of Graduate Students and Postdoctorates in Science and Engineering. The majority of these data are publicly available on institutional websites, and some were obtained informally by e-mail from HR contacts or postdoctoral offices. Click here for additional data file. Copyright: © 2017 Bankston A and McDowell GS 2017 Data associated with the article are available under the terms of the Creative Commons Zero "No rights reserved" data waiver (CC0 1.0 Public domain dedication).

Minimum wage information from all U.S. states (correct at time of writing) This information was gathered from comprehensive sources, and showed changes mainly for New York and California. Click here for additional data file. Copyright: © 2017 Bankston A and McDowell GS 2017 Data associated with the article are available under the terms of the Creative Commons Zero "No rights reserved" data waiver (CC0 1.0 Public domain dedication).

Five of the institutions which initially cancelled plans to raise salaries have since largely reversed their decision to do so, with some exceptions made. These are the University of Michigan, the University of Illinois, Brigham and Women’s (Boston, MA), Iowa State University and Massachusetts General Hospital.

## Conclusions

### Discussion of the data collected

We approached the data collection by first assuming that all institutions would have postdoc salary information transparently and publicly available, whereas of course there was great variation in the institutions publishing this information on the web or responding to queries for FLSA compliance information, and this did not seem to be linked to the size of the institution, which hindered our ability to gather the data. We have observed a greater demand for increased transparency about postdoc salaries, and benefits, and the Boston Postdoc Association is one example of an organization that has collected and published this data themselves since we began our efforts (see http://bostonpostdocs.org/advocacy/benefits/, ( Cijsouw *et al.*, 2017)).

As we have discussed the difficulties with using the NSF data on postdoc numbers elsewhere ( Pickett *et al.*, 2017), it is understood that these numbers likely have some degree of variation. In contrast to the Council on Governmental Relations report of August 2016, we saw a very striking difference in institutional plans. Decisions were expected to have been made at this time at 97% of institutions, whereas we were able to find only 43% of them had done so one month before the FLSA implementation date. The ratio of institutions planning to raise salaries to those planning to track hours was 3:1 in the report, whereas our data show the ratio closer to 9:1. Why are these numbers so different? It is possible that the sample surveyed was biased towards the hours-tracking institutions, but what seems more likely is that as institutions investigated the legal ramifications of tracking hours, and reconciled these with the culture of academe, they likely revised their plans in recognition that postdocs could not comfortably fit their work into this system in an affordable manner, or that FLSA violations would be extremely likely.

### Effects on postdocs after the injunction

The injunction granted in November 2016 left postdocs feeling confused and disposable to the scientific enterprise. Postdoc reactions were documented by various sources ( Anon, n.d.) in which postdocs gave voice to their sense of feeling undervalued by the academic system, particularly after the injunction. We began attempting to collect salaries of individual postdocs at public institutions to assess the current state of postdoctoral salaries. Our preliminary analysis shows a sizeable portion of reported full-time postdoctoral annual salaries reaching as low as the current legal minimum of $23,660. There are discrepancies in salary reporting, particularly if salaries are paid from multiple sources. This effort will be discussed in future work. The difficulty we have faced in attempting to obtain basic information from institutions gives us cause for concern that the general standard of postdoc administration at institutions is worse even than currently supposed.

### Next steps: Individual states implementing updates to the FLSA

One recent development is that the new U.S. administration solicited feedback on proposed updates to the FLSA ( Department of Labor, 2017), although it may be unlikely to reproduce some of the effects desired by the previous administration. The Union of Auto Workers submitted comments specifically discussing postdocs ( https://www.regulations.gov/document?D=WHD-2017-0002-139165 and https://www.regulations.gov/document?D=WHD-2017-0002-140022).

However, some U.S. states are going ahead with updates from their own Departments of Labor. New York and California in particular are states where postdocs may be affected within the next 1–2 years. We have gathered minimum wage information for all U.S. states ( Dataset 2).

### Issues encountered so far

***Were postdocs on fellowships FLSA exempt?*** Brown, Brandeis and Rutgers universities suggested that postdocs paid on training grant/fellowship stipends would be FLSA exempt, and that they would not be mandating raises in the salaries of postdocs on stipends below the exemption level. No responses to requests for their legal justification was ever received. Nevertheless, the position of Brown University was stated explicitly as follows:

“ *Postdoctoral fellows are defined as non-employees, paid by stipend rather than salary, and are thus not covered by the FLSA* ( Brown University, 2016).”

The position of Brandeis University was stated publicly on their website as follows (with info that salaries need to be raised for postdoctoral associates, but with no mention of fellows):

“Postdoctoral Fellows come to Brandeis to further their scholarly competence, with fellowship aid through sources other than the NRSA. These sources may be federal or non-federal. Appointments are usually for one semester or more and are renewable, based upon the terms and conditions of the individual award. Postdoctoral Fellows are trainees and do not provide services to the University, and are not considered to be employees. A Postdoctoral Fellow is eligible to be appointed as a Postdoctoral Associate after the term of the Postdoctoral Fellowship has ended ( Brandeis University, 2016).”

Postdoctoral fellows are often not considered employees by institutions, as they are not paid by the institution. However, reading the directions from the Department of Labor, that is not the same as being recognized as exempt from the FLSA. A postdoc is federally recognized as both a trainee and an employee (Code of Federal Regulations, Title 2, part 200.400(f) ( U.S. Government, 2014)) and unless they are in a primarily teaching role, all postdocs come under the FLSA (see Overtime Final Rule and Higher Education ( United States Department of Labor, 2016c)). In addition, the Department of Labor defines what “employ’ means in the context of the FLSA:

“ *The FLSA defines the term “employ” to include the words “suffer or permit to work”. Suffer or permit to work means that if an employer requires or allows employees to work, the time spent is generally hours worked* ( United States Department of Labor, n.d.).”

The “employer” is the institution that “suffers [someone] to work,” so institutions are the ones responsible for ensuring FLSA compliance. Postdoctoral fellows are “permitted” to work at the institution. In combination with further guidance from the Department of Labor on independent contractors, the closest possible analogy to postdoctoral fellows ( United States Department of Labor, 2014), it was our understanding that if postdoctoral fellowships did not pay stipends above the new FLSA minimum, the employer was still responsible for making sure the salary of employees was supplemented up to federal standards. This was also the understanding made clear to the NIH by the Department of Labor, driving the increase in the NRSA levels ( National Institutes of Health, 2016). What matters from the point of view of the Department of Labor is not where the money comes from, but what a person is doing at an institution that “suffers or permits them to work” there. Fellows and non-fellows alike carry out work of identical nature, and so both fall under the FLSA.

This may become part of the larger conflict in the debate over whether postdocs are trainees or employees, and what services (including intellectual property) they do or do not provide to the university ( Haak, 2002a; Haak, 2002b; Haak, 2002c). This aspect may aggravate existing issues with postdoctoral fellowships, as recipients already face losing benefits ( Gaval, 2014), or dealing with tax complications such as imputed income tax ( National Postdoctoral Association, n.d.; University of California San Francisco, 2015). Elsewhere we have discussed changes that may be taking place to postdoctoral benefits, such as reductions in fringe rates, as a similar but separate effect of the FLSA implementation at institutions. For example, postdocs at institutions such as the University of Alabama Birmingham actually stood to lose money overall, if they had a family, as they were now required to cover 25% of their healthcare as a result of institutional policy changes ( Future of Research, 2016a).

In general, there were a number of loopholes that institutions were actively exploiting in an apparent attempt to recover or reduce costs, illustrating the need to closely monitor institutional activities with respect to how they deal with those undertaking training at their institutions.

***If violations to the FLSA ruling occurred, would they have been reported?*** One question that occupied some discussion about the FLSA was whether violations, such as directing employees to give false reports on timesheets, would actually be reported. This is now a relatively moot point; however the Department of Labor has advice on how to report these violations ( United States Department of Labor, 2016d) and points out that there is a three year statute of limitations, and reporting is completely confidential until the point of allegation being pursued; at that point, the person deals with the Department of Labor and not the institution. In addition, it is illegal for employers to take action against employees based on reporting of violations, and their immigration status will not be investigated.

Comparisons were made between the perceived lack of reporting violations in tracking hours during medical residency, and what could occur with the new postdoc system in academia. We use this comparison here to illustrate why reporting hours could have been more common in academic science. First, medical residents are exempt from the FLSA, so a different system of salary reporting exists to begin with. Medical residents can have up to an additional ~$250,000 debt for tuition, compared to the relatively lower student debts in the academic path, as well as the cultural eschewing of financial gain, and perhaps have more “skin in the game”. The bottleneck in the medical system is often getting into residency, from where job certainty is much higher than in academia, where most postdocs end up leaving academia despite a high interest in staying ( Sauermann & Roach, 2016). In addition, medical residents may consider that reporting hours could actually harm their own training and the training of others, whereas whether many postdocs actually receive training is of great concern in academe ( Pickett *et al.*, 2015), and this certainly rarely happens to a cohort of postdocs at once (unlike medical residency), and perhaps the perceived harm to that training may be seen as minimally impacting them. This perception, combined with the 3 year statute of limitations, makes “burning bridges” a much stronger possibility for postdocs.

If a violation of the FLSA is reported, it seems that the burden of proof is on the institution to counter the evidence from the complaint (discussed in ( American Council on Education, 2016)). There were many common false assumptions being discussed in academia about how federal labor law could be implemented ( Reynolds & Rudnick, 2010). This made it very interesting that a number of institutions seemed willing to allow departments, or even individual PIs, with poor understanding of federal labor laws, to decide on how to administer postdoctoral salaries at the institutional level.

***Moving towards greater transparency in postdoc salaries.*** Our goal in presenting these data is to increase transparency about the postdoc position, in terms of their administration and benefits, in a similar manner to a call for transparency in career outcomes ( Polka *et al.*, 2015). Here, we have presented our impression of the information currently available to postdocs about how salaries may broadly have changed due to updates to the FLSA. We are currently analyzing postdoctoral salaries as of December 1, 2016 at a number of public institutions, and plan to carry out annual samplings of the salaries of postdocs at U.S. institutions going forward, as far as it is possible given the barriers to collecting this data.

***Effects of the FLSA updates which did not come to pass.*** We previously speculated on the possible effects that the updates to the FLSA may have had on postdocs - tracking hours ran counter to academic culture, and the notion of the postdoc being a “trainee”, or someone in a mentored environment developing research independence. This is also in contrast to behaviors learned in graduate school and the working culture of the faculty positions to which postdocs are meant to be directed. Analyzing data, writing and reading papers and carrying out other job related duties of the postdoc are often performed during nights and weekends, while away from the lab. How would those hours have been tracked to everyone’s satisfaction? The relatively small numbers of institutions tracking hours for postdocs suggested that these issues were appreciated by many institutions.

In addition, there is now a clear differential landscape of institutions who raised or did not raise postdoc salaries, which creates a wide discrepancy and may influence the market for attracting postdocs, which both potential postdocs and the principal investigators looking to hire them should be aware of.

There are still many questions that this change in the postdoctoral salary landscape raises. How will this affect smaller institutions? Will there be a drop in new postdoc hires as they become more expensive, will postdocs be paid out of grants in the long run, or will some other institutional mechanisms be employed? Given that international postdocs are generally presumed to earn less, will they continue to do so, or will salary increases at institutions affect the demographics of the postdoc population? How many postdocs are about to lose their jobs (bearing in mind that wider labor market analysis found no drop in employment levels over seven decades of minimum wage increases ( Sonn & Lathrop, n.d.))? How many postdocs have to shorten their current positions? Will junior faculty bear the brunt, will mid-career researchers be most strained, or will tenured professors be more likely to cut postdocs loose? Will institutions look to increase admission of graduate students, to keep up the labor at the bench? Will there be a shift to more postdocs on particular training mechanisms or fellowships, where the salary is provided, and less on other types of fellowships or research grants?

Perhaps most importantly, will the research enterprise start acting on salary recommendations for postdocs in a more timely fashion? The academic system will survive this modernization, and in the long term the likely decrease of the postdoctoral population may be a necessary cap on the expanding “trainee” population. This will make the research enterprise more sustainable by limiting the number of postdocs which the system can support. The FLSA ruling change could have been handled far better by the system in favor of postdocs. Postdocs have traditionally been severely underpaid, and have also had other significant burdens placed on them - not limited to long working hours with little reward, and work-life balance issues.

Unfortunately, recommendations to raise postdoc salaries have been ignored for a long time by universities. The salary raise imposes changes on the system at universities, but these changes could have been implemented more gradually, and with less pain inflicted on postdoctoral researchers than they are now. This would ensure that postdocs are treated more fairly, or at least dealt with as the highly-trained PhD scientists that they are. How to correctly deal with the administration of postdocs at institutions has been in discussion over decades ( Curtis, 1969), and raising postdoc salaries has previously been advocated for. Hopefully this study will serve as a call to action in terms of how to deal with other issues affecting the research enterprise in the future. Will other recommended changes within the research enterprise be made by the deliberate action of scientists and administrators, or will they have to be imposed by federal statutes? We hope that the abrupt nature of the FLSA revision serves as a call to redouble efforts for academia to become the driver, rather than the subject, of change.

## Data availability

The data referenced by this article are under copyright with the following copyright statement: Copyright: © 2017 Bankston A and McDowell GS

Data associated with the article are available under the terms of the Creative Commons Zero "No rights reserved" data waiver (CC0 1.0 Public domain dedication). 

F1000Research: Dataset 1. Status of postdoc policies prior to and following the injunction against updates to the FLSA, 10.5256/f1000research.10086.d177471 ( Bankston & McDowell, 2017a)

F1000Research: Dataset 2. Minimum wage information from all U.S. states (correct at time of writing), 10.5256/f1000research.10086.d177472 ( Bankston & McDowell, 2017b)
