# Supplementary material for: Monitoring the compliance of the academic enterprise with the Fair Labor Standards Act
Source: F1000Res. 2017 Sep 28;5:2690. Originally published 2016 Nov 17. [Version 2] doi: 10.12688/f1000research.10086.2 (PMC5130071; doi:10.12688/f1000research.10086.2)
Supplement: Minimum wage information from all U.S. states (correct at time of writing) — This information was gathered from comprehensive sources, and showed changes mainly for New York and California. [file f1000research-5-13681-s0001.tgz › 1e2e0c83-cc57-44e0-b4b4-8fd3ef0d2f95_Dataset_2_Minimum_wage_information_from_all_U.S._states.pdf]

Monitoring the compliance of the academic enterprise with the Fair Labor Standards Act
Adriana Bankston, Gary S. McDowell, 2017

Dataset 2. Minimum wage information from all U.S. states (correct at time of writing). This information was gathered from comprehensive sources, and showed changes mainly for New York and California.

Alabama: "Alabama has not established a state minimum wage rate. Because there is not an Alabama minimum wage and most employers and employees in Alabama are subject to the federal Fair Labor Standards Act, the minimum wage set forth in that law would typically apply."

Alaska: "Alaska's current minimum wage is $9.80. Beginning on January 1, 2017, Alaska's minimum wage is pegged to inflation and will increase each year by the increase in the Consumer Price Index for all urban consumers for the Anchorage metropolitan area, compiled by the Bureau of Labor Statistics, United States Department of Labor, rounded to the nearest one cent. If at any time Alaska's minimum wage is less than $1.00 more than Alaska's minimum wage, Alaska's minimum wage will adjust to be $1.00 more than the federal minimum wage and any future minimum wage adjustments will be based on the new minimum wage. Alaska employers must also comply with federal minimum wage laws, which currently sets the federal minimum wage at $7.25. See FLSA: Minimum Wage."

Arizona: "Arizona's current minimum wage is $10.00. Arizona minimum wage will increase in subsequent years as follows:

- January 1, 2018: $10.50
- January 1, 2019: $11.00
- January 1, 2018: $12.00

Beginning on January 1, 2021, the minimum wage will increase by the percentage increase in the cost of living. AZ Industrial Commission – Frequently Asked Questions (FAQs) About Minimum Wage and Earned Paid Sick Time

Arizona employers must also comply with federal minimum wage laws, which currently sets the federal minimum wage at $7.25. See FLSA: Minimum Wage.

If an employer chooses to pay employees minimum wage, the employer must pay those employees in accordance with the minimum wage law, either federal or state, that results in the employees being paid the higher wage. In most instances in Arizona, the Arizona minimum wage will apply as it generally guarantees a higher wage rate for employees than federal law."

Arkansas: "Arkansas' current minimum wage is $8.50. AR Code 11-4-210. Arkansas' minimum wage requirement applies only to employers with four or more employees unless an exception or exemption applies. AR Code 11-4-203

Arkansas employers must also comply with federal minimum wage laws, which

currently sets the federal minimum wage at $7.25. See FLSA: Minimum Wage.

If an employer chooses to pay employees minimum wage, the employer must pay those employees in accordance with the minimum wage law, either federal or state, that results in the employees being paid the higher wage. In most instances in Arkansas, the federal minimum wage law will apply as it generally guarantees a higher wage rate for employees than state law."

California: "California's current minimum wage is $10.50 for employers with 26 or more employees and $10.00 for employers with 25 or fewer employees. CA Labor Code 1182.12; CA Minimum Wage Order (MW-2017). California's minimum wage will increase over the next several years as follows:

Employers with 26 or more employees:

- January 1, 2018 – $11.00
- January 1, 2019 – $12.00
- January 1, 2020 – $13.00
- January 1, 2021 – $14.00
- January 1, 2022 – $15.00

Employers with 25 or fewer employees:

- January 1, 2018 – $10.00
- January 1, 2019 – $11.00
- January 1, 2020 – $12.00
- January 1, 2021 – $13.00
- January 1, 2022 – $14.00
- January 1, 2023 – $15.00

Beginning on January 1, 2023, for employers with 26 or more employees and January 1, 2024, for employers with 25 or fewer employees, California will increase its minimum wage by the lesser of:

- 3.5 percent and
- the rate of change in the averages of the most recent July 1 to June 30, inclusive, period over the preceding July 1 to June 30, inclusive, period for the United States Bureau of Labor Statistics nonseasonally adjusted United States Consumer Price Index for Urban Wage Earners and Clerical Workers (U.S. CPI-W) rounded to the nearest ten cents ($0.10).

The California Director of Finance is responsible for determining the change in minimum wage based on the calculations discussed above by August 1 of the year before the change will take place. Additional rules apply that may affect California's minimum wage calculation.

California employers must also comply with federal minimum wage laws, which currently sets the federal minimum wage at $7.25. See FLSA: Minimum Wage.

If an employer chooses to pay employees minimum wage, the employer must pay those employees in accordance with the minimum wage law, either federal or state,

that results in the employees being paid the higher wage. In most instances in California, the California minimum wage will apply as it generally guarantees a higher wage rate for employees than federal law."

California: (another source) "The state minimum wage is scheduled to increase in phases between 2017 and 2022" - as it's linked to the increasing minimum wage, will hit $62,400 by 2022. It doesn't apply to public employees (including UC) but applies to all others.

Colorado: "Colorado's current minimum wage is $9.30. Colorado Department of Labor: Minimum Wage Colorado Constitution Article XVIII, Section 15, requires that the state's minimum wage be increased annually for inflation. The inflation adjustment is based on changes in the Consumer Price Index for All Urban Consumers (CPI-U), All Items, for the Denver-Boulder-Greeley combined metropolitan statistical area as published by the United States Bureau of Labor Statistics (BLS). The changes to the minimum wage based on the cost of living adjustment will take effect on January 1 of each year. CO Constitution, Art. XVIII, Section 15; CO Dept. of Labor and Employment – Minimum Wage."

Connecticut: "Connecticut's current minimum wage is $10.10. CT Public Act No. 14-1. Connecticut's minimum wage law provides that its minimum wage will increase to be one-half of one percent more than the federal minimum wage when it increases, rounded to the nearest whole cent. Any increase to Connecticut's minimum wage resulting from an increase to the federal minimum wage rate takes effect on the same day as the federal rate change. CT Statutes 31-58(j)

Connecticut employers must also comply with federal minimum wage laws, which currently sets the federal minimum wage at $7.25. See FLSA: Minimum Wage. If an employer chooses to pay employees minimum wage, the employer must pay those employees in accordance with the minimum wage law, either federal or state, that results in the employees being paid the higher wage. In most instances in Connecticut, the Connecticut minimum wage will apply as it generally guarantees a higher wage rate for employees than federal law."

Delaware: "Delaware's current minimum wage is $8.25. By law, if the federal minimum wage set forth in the Fair Labor Standards Act increases to be greater than the minimum wage set by Delaware minimum wage laws, Delaware's minimum wage will automatically increase to match the federal rate. DE Statutes 19-902(a) Delaware employers must also comply with federal minimum wage laws, which currently sets the federal minimum wage at $7.25. See FLSA: Minimum Wage.

District of Columbia: "The District of Columbia's current minimum wage is $11.50. The minimum wage will increase in subsequent years as follows:

- July 1, 2017 – $12.50
- July 1, 2018 – $13.25
- July 1, 2019 – $14.00
- July 1, 2020 – $15.00

Beginning July 1, 2021, the District of Columbia will increase its minimum wage annually in proportion to the annual average increase in the Consumer Price Index for All Urban Consumers in the Washington Metropolitan Statistical Area for the

preceding 12 months. DC Fair Shot Minimum Wage Amendment Act of 2016 (B21-712)

Employers of security officers working office buildings must pay the security officers wages, or any combination of wages and benefits, that are not less than the combined amount of the minimum wage and fringe benefit rate for the guard 1 classification established by the United States Secretary of Labor pursuant to the Service Contract Act of 1965, approved October 22, 1965 (79 Stat. 1034; 41 U.S.C. 351). D.C. Code 32-1003(h)

An individual person is considered to be employed in the District of Columbia when:

- the individual regularly spends more than 50% of his or her working time in the District of Columbia; or
- the individual's employment is based in the District of Columbia and the individual regularly spends a substantial amount of his or her working time in the District of Columbia and not more than 50% of their working time in any particular state. D.C. Code 32-1003(h)"

Florida: "Florida's current minimum wage is $8.10. FL Minimum Wage Florida minimum wage laws are found in Florida's constitution and required an annual review of its minimum wage. Changes to the rate must be determined by September 30 of each year and take effect on January 1 of the following year. Any changes to Florida's minimum wage must be based on changes in the consumer price index. FL Constitution, Art. X, Sec. 24; FL Stat. 448.110.

Florida employers must also comply with federal minimum wage laws, which currently sets the federal minimum wage at $7.25. See FLSA: Minimum Wage.

If an employer chooses to pay employees minimum wage, the employer must pay those employees in accordance with the minimum wage law, either federal or state, that results in the employees being paid the higher wage. In most instances in Florida, Florida minimum wage laws will apply as it generally guarantees a higher wage rate for employees than federal law."

Georgia: "Georgia's current minimum wage is $5.15. Georgia's minimum wage law does not apply to employers who are subject to the federal Fair Labor Standards Act when the federal Fair Labor Standards Act requires employers to pay a minimum wage greater than the Georgia's minimum wage. O.C.G.A. 34-4-3(c)

Georgia minimum wage laws also do not apply to the following employers:

- employers with sales of $40,000 per year or less;
- employers with five (5) employees or less
- employers of domestic employees;
- employers who are farm owners, sharecroppers, or land renters;

O.C.G.A. 34-4-3."

Hawaii: "Hawaii's current minimum wage is $9.25. HI Statute 387-2

Hawaii's minimum wage will increase to $10.10 on January 1, 2018. HI Wage Standards Division – Minimum Wage and Overtime

Hawaii employers must also comply with federal minimum wage laws, which currently sets the federal minimum wage at $7.25. See FLSA: Minimum Wage."

Idaho: "Idaho's current minimum wage is $7.25. ID Statute 44-1502.

Idaho employers must also comply with federal minimum wage laws, which currently sets the federal minimum wage at $7.25. See FLSA: Minimum Wage."

Illinois: "Illinois' current minimum wage is $8.25. IL Statute 820-105/4

Illinois employers must also comply with federal minimum wage laws, which currently sets the federal minimum wage at $7.25. See FLSA: Minimum Wage.

If an employer chooses to pay employees minimum wage, the employer must pay those employees in accordance with the minimum wage law, either federal or state, that results in the employees being paid the higher wage. In most instances in Illinois, the Illinois minimum wage will apply as it generally guarantees a higher wage rate for employees than federal law."

Indiana: "Indiana's current minimum wage law is $7.25. IN Code 22-2-2-4(h)
Indiana employers must also comply with federal minimum wage laws, which currently sets the federal minimum wage at $7.25. See FLSA: Minimum Wage."

Iowa: "Iowa's current minimum wage is $7.25. IA Code 91D.1(1)(a)
Iowa's minimum wage requirements do not apply to employers that have an annual gross volume of sales made or business done that is less than $300,000, not including any excise tax at the retail level that are recorded separately, except the following which are not subject to a minimum sales or business threshold:

- employer that launder, clean, or repair clothing or fabrics;
- employers that engage in construction or reconstruction;
- hospitals or other institutions primarily engaged in the care of the sick, aged, or mentally ill, including institutions that treat individuals with symptoms of mental illness who reside at the premises of the institution;
- schools, including those for individuals with physical disabilities or gifted children, preschools, elementary and secondary schools, and institutions of higher education; and
- public agencies.

IA Code 91D.1(2)

Employers subject to the federal Fair Labor Standards Act must pay employees the federal minimum wage rate if it is greater than the Iowa rate. If the federal rate is ever lower than the Iowa rate, employers must pay employees the Iowa rate. IA Code 91D.1(1)(b)"

Kansas: "Kansas' current minimum wage is $7.25. KS Statute 12-1203
Kansas minimum wage laws do not apply to employers who are covered by the federal Fair Labor Standards Act. KS Statute 12-1202(d)"

Kentucky: "Kentucky's current minimum wage is $7.25. Should the federal minimum hourly wage increase, the minimum hourly wage in Kentucky will also increase to the same amount.
KY Statute 337.275(1)."

Louisiana: "Louisiana has not establish a state minimum wage rate. Louisiana law prohibits any governmental subdivision of the state from creating a minimum wage for employees. LA Statute 23:642. Employers would be required to pay employees the federal minimum wage as required by the Fair Labor Standards Act. Currently, the federal minimum wage is $7.25."

Maine: "Maine's current minimum wage is $9.00.
Maine's minimum wage will increase as follows over the coming years:

- January 1, 2018: $10.00
- January 1, 2019: $11.00
- January 1, 2020: $12.00

Beginning on January 1, 2021, the minimum wage will increase each year consistent with the percentage increase for the prior year in the Consumer Price Index for Urban Wage Earners and Clerical Workers, CPI-W, for the Northeast Region, or its successor index, as published by the United States Department of Labor, Bureau of Labor Statistics or its successor agency, with the amount of the minimum wage increase rounded to the nearest multiple of $0.05.

If the federal minimum wage set forth in the Fair Labor Standards Act increases to be greater than Maine's minimum wage, the state's minimum wage will increase to match the federal wage. Any increases to Maine's minimum wage triggered by changes in the federal minimum wage shall go into effect on the same day but shall not exceed $1 above the state's current rate. ME Statute 664."

Maryland: "Maryland's current minimum wage is $8.75, except in Prince George's and Montgomery counties where the minimum wage is $10.75.
Maryland's minimum wage will increase over the next several years as follows:

- July 1, 2017: $9.25
- July 1, 2018: $10.10

MD Statutes, Labor and Employment Article 3-413; Maryland Minimum Wage and Overtime Law

The minimum wage for Prince George's and Montgomery counties will increase on October 1, 2017, to $11.50.

Minimum Wage and Overtime Law in Prince George's County; Minimum Wage and Overtime Law in Montgomery County"

Massachusetts: "Massachusetts' current minimum wage is $11.00.
MA Laws 151-1; MA Dept. of Labor and Workforce Dev. – Minimum Wage Letter

Massachusetts minimum wage laws require that the state's minimum wage remains at least fifty (50) cents higher than the federal minimum wage set forth in the Fair Labor Standards Act. MA Laws 151-1.

Massachusetts employers must also comply with federal minimum wage laws, which currently sets the federal minimum wage at $7.25. See FLSA: Minimum Wage.

If an employer chooses to pay employees minimum wage, the employer must pay those employees in accordance with the minimum wage law, either federal or state, that results in the employees being paid the higher wage. In most instances in Massachusetts, the Massachusetts minimum wage will apply as it generally guarantees a higher wage rate for employees than federal law."

Michigan: "Michigan's current minimum wage is $8.90.
Michigan's minimum wage will increase to $9.25 on January 1, 2018.

MI Laws 408.414; MI Wage and Hour Program – Workforce Opportunity Wage Act

Beginning April 1, 2019, and every year thereafter, Michigan will increase its minimum wage to reflect the average annual percentage change in the consumer price index for the most recent 5-year period for which data is available, although the change to the minimum wage cannot exceed 3.5% per year. Additionally, the minimum wage will not increase in any year where the unemployment rate is 8.5% or greater for the year preceding the year in which the increase was to take effect. The state treasure will be responsible for calculating the change in the consumer price index and will rely on the most comprehensive index of consumer prices available for the Midwest region from the U.S. Department of Labor, Bureau of Labor Statistics. The Michigan Department of Licensing and Regulatory Affairs, Wage and Hour Division, must post the adjusted minimum wage on its website by February 1 of the year the change will become effective. The cost of living increase will not take effect if the unemployment rate in Michigan is 8.5% or more in the prior year. MI Law 408.414

Michigan minimum wage laws apply to employers who employ two or more employees over the age of 16 at the same time within a calendar year. MI Law 408.382; MI Law 408.384

Michigan employers must also comply with federal minimum wage laws, which currently sets the federal minimum wage at $7.25. See FLSA: Minimum Wage.

If an employer chooses to pay employees minimum wage, the employer must pay those employees in accordance with the minimum wage law, either federal or state, that results in the employees being paid the higher wage. In most instances in Michigan, the Michigan minimum wage will apply as it generally guarantees a higher wage rate for employees than federal law."

Minnesota: "Minnesota minimum wage laws set different minimum wages for large employers and small employers, as discussed below.

Large employers

Minnesota's current minimum wage for large employers is $9.50.

A "large" employer is an enterprise with at least $500,000 in gross annual sales made or business made (exclusive of excise taxes at the retail level that are separately stated) and that is not otherwise exempt from Minnesota's Fair Labor Standards Act. MN Statute 177.24(1)(a)

Small employers

Minnesota's current minimum wage for small employers is $7.75.

A"small" employer is an enterprise with no more than $500,000 in gross annual sales made or business made (exclusive of excise taxes at the retail level that are separately stated) and that is not otherwise exempt from Minnesota's Fair Labor Standards Act. MN Statute 177.24(1)(a)

Cost of living increases beginning January 2018

Beginning in August 2017, Minnesota minimum wage law requires the Department of Labor and Industry to conduct an annual review each August of its minimum wage and increase the minimum wage by the lesser of:

- 2.5 percent, or
- the percentage increase in the rate of inflation, as measured by the implicit price deflator, national data for personal consumption expenditures as determined by the United States Department of Commerce, Bureau of Economic Analysis in the 12-month period immediately preceding the August in which the review is conducted or during the most recent period for which the information is available.

Any change to the minimum wage takes effect on January 1 of the following year, with the first cost of living adjustment taking effect on January 1, 2018. In certain circumstances and prior to September 30 in a given year, the Minnesota Department of Labor and Industry may choose not to increase the minimum wage the following year. It may refuse to increase the minimum wage in a given year only if it finds, after consultation with the Department of Management and Budget, that the leading economic indicators indicate the potential for a substantial downturn in the state's economy. It must also consider the ratio of the rate of the calculated change to the minimum wage to the rate of change in the state median income over the same period of time. Prior to implementing the decision not to increase the minimum wage, the

Department of Labor and Industry must conduct a public hearing and allow for written comments prior to the hearing and for twenty (20) days after. The Department of Labor and Industry may make adjustments to the minimum wage in subsequent years to make up for the lack of an increase in prior years. MN Statute 177.24(1)(f)

Minnesota employers must also comply with federal minimum wage laws, which currently sets the federal minimum wage at $7.25. See FLSA: Minimum Wage.

If an employer chooses to pay employees minimum wage, the employer must pay those employees in accordance with the minimum wage law, either federal or state, that results in the employees being paid the higher wage. In most instances in Minnesota, the state minimum wage will apply as it generally guarantees a higher wage rate for employees than state law."

Mississippi: "Mississippi has not established a state minimum wage rate. Because most employers and employees in Mississippi are subject to the federal Fair Labor Standards Act, the minimum wage set forth in that law would typically apply. Currently, the federal minimum wage is $7.25."

Missouri: "Missouri's current minimum wage is $7.70. See MO Dept. of Labor: Minimum Wage
Missouri minimum wage laws require an annual review of its minimum wage and the minimum wage must be increased by the percentage the cost of living has changed from the prior July to the July in the year the review is conducted. The review is conducted on September 30 of each year and the cost of living change is based on the Consumer Price Index for Urban Wage Earners and Clerical Workers (CPI-W). Any change to the minimum wage takes effect on January 1 of the following year. MO Statute 290.502(2)

An employer may take a credit against its obligation to pay minimum wage for the fair market value of goods and services received and retained by employees so long as the employees voluntarily receive the goods and services and they are for the private benefit of the employees. The method of valuing good and services is set forth in Missouri Regulation 8:30-4.050. MO Statute 290.512(2)

Missouri employers must also comply with federal minimum wage laws, which currently sets the federal minimum wage at $7.25. See FLSA: Minimum Wage.

If an employer chooses to pay employees minimum wage, the employer must pay those employees in accordance with the minimum wage law, either federal or state, that results in the employees being paid the higher wage. In most instances in Missouri, the Missouri minimum wage will apply as it generally guarantees a higher wage rate for employees than federal law."

Montana: "Montana's current minimum wage is $8.15, for employers who have more than $110,000 in annual gross sales. MT Dept. of Labor & Industry: Minimum Wage
For employers who have gross annual sales of $110,000 or less, the minimum wage rate is $4.00. MT Code 39-3-409(3)

Montana law requires an annual review of its minimum wage and the minimum wage must be increased by the percentage the cost of living has changed from the prior August to the August in the year the review is conducted. The cost of living change is based on the consumer price index. Any change to the minimum wage takes effect on January 1 of the following year. MT Code 39-3-409(2)

Montana employers must also comply with federal minimum wage laws, which currently sets the federal minimum wage at $7.25. See FLSA: Minimum Wage.

If an employer chooses to pay employees minimum wage, the employer must pay those employees in accordance with the minimum wage law, either federal or state, that results in the employees being paid the higher wage. In most instances in Montana, the Montana minimum wage will apply as it generally guarantees a higher wage rate for employees than federal law."

Nebraska: "Nebraska's current minimum wage is $9.00. Nebraska Minimum Wage Increase, Initiative 425 (2014)
Nebraska minimum wage laws do not apply to employers with fewer than 4 employees. NE Statute 48-1202(2)

Nebraska employers must also comply with federal minimum wage laws, which currently sets the federal minimum wage at $7.25. See FLSA: Minimum Wage.

If an employer chooses to pay employees minimum wage, the employer must pay those employees in accordance with the minimum wage law, either federal or state, that results in the employees being paid the higher wage."

Nevada: "Nevada's current minimum wage is $7.25 for employees who are offered qualifying health benefits. It is $8.25 for employees who have not be offered qualifying health benefits. NV Constitution, Art. 15, Sec. 16.; NV Statute 608.250; NV Admin. Code 608.100; NV Labor Comm.: Minimum Wage Bulletin.

Nevada's constitution requires an annual review of its minimum wage. The minimum wage must be increased by the percentage the cost of living has changed from December 31 in the given you over the level as of December 31, 2004, up to three (3) percent. The cost of living change is based on the Consumer Price Index published by the Bureau of Labor Statistics, U.S. Department of Labor. Any change to the minimum wage will be announced by the Governor or a designated state agency by April 1 of a given year and the change will take effect July 1 of that same year. Moreover, Nevada's minimum wage may not be lower than the federal minimum wage and will be adjusted accordingly when necessary. A change in Nevada's minimum wage due to an increase in the federal minimum wage may take the place of the annual cost of living increase. NV Constitution, Art. 15, Sec. 16

A qualifying health insurance plan that permits an employer to pay the lower minimum wage rate must:

- cover all categories of health care expenses an employee would generally be able to deduct from their federal income tax return pursuant to 26 USC 213 and any related regulations if they paid for the expenses themselves; or
- provide health benefits pursuant to a Taft-Hartley trust which:
  - if formed pursuant to 29 USC 186(c)(5); and
  - qualifies as an employee welfare benefit plan under:
    - Internal Revenue Service guidelines; or
    - the Employee Retirement Income Security Act (ERISA)
- be made available to the employee and any dependents, which is evidenced by:
  - the employer maintains contracts for health insurance for the class of employees being paid the lower minimum wage, subject only to conditions required to complete coverage that are applicable to similarly situated employees in the same class; and
  - employees do not have to wait more than six (6) months to be eligible to participate in the health insurance plan.
  - require that employees pay no more than 10 percent of their gross taxable income for premiums.

NV Admin Code 608.102

An employee may voluntarily decline coverage for qualifying health benefits and remain eligible for the lower minimum wage. However, the employer must maintain documentation that the employee has declined coverage and the employer may not require any employee decline coverage as a term or condition of employment. NV Admin Code 608.106

If an employer requires new hires to work for a period of time before becoming eligible to participate in a qualifying health benefits plan, the employer must pay the new hire the higher minimum wage until the employee's waiting period ends and the employee becomes eligible to participate in the plan. NV Admin Code 608.108"

New Hampshire: "New Hampshire's current minimum wage is $7.25.
New Hampshire law sets its minimum wage to be the same as federal minimum wage set forth in the Fair Labor Standards Act. NH Statute 279:21.

New Hampshire employers must also comply with federal minimum wage laws, which currently sets the federal minimum wage at $7.25. See FLSA: Minimum Wage.

If an employer chooses to pay employees minimum wage, the employer must pay those employees in accordance with the minimum wage law, either federal or state, that results in the employees being paid the higher wage."

New Jersey: "New Jersey's current minimum wage is $8.44. NJ Constitution Art. I, Section 23; NJ Dept. of Labor and Workforce Dev.: Wage and Hour Law Abstract
New Jersey's Constitution requires an annual review of its minimum wage. The minimum wage must be increased by the percentage the cost of living has changed from the prior September 30 to September 30 in the year the review is conducted. The cost of living change is based on consumer price index for all urban wage earners and clerical workers (CPI-W) published by the federal government. Any change to the

minimum wage takes effect on January 1 of the following year. Additionally, if the federal minimum wage is raised to a level higher than that of New Jersey, New Jersey's minimum wage automatically increases to the higher federal rate and all subsequent cost of living increases to New Jersey's minimum wage will be based off of the new rate. FLSA: Minimum Wage.

If an employer chooses to pay employees minimum wage, the employer must pay those employees in accordance with the minimum wage law, either federal or state, that results in the employees being paid the higher wage. In most instances in New Jersey, the New Jersey minimum wage will apply as it generally guarantees a higher wage rate for employees than federal law."

New Mexico: "New Mexico's current minimum wage is $7.50. NM Statute 50-4-22. As of January 1, 2014, the minimum hourly wage for Albuquerque is $8.60. It is $7.60 if the employer provides healthcare and/or childcare benefits to the employee during any pay period and the employer pays an amount for these benefits equal to or in excess of an annualized cost of $2,500.00.Albuquerque Minimum Wage.

Effective March 1, 2013, Santa Fe minimum wage is $10.66 per hour.

New Mexico employers must also comply with federal minimum wage laws, which currently sets the federal minimum wage at $7.25. See FLSA: Minimum Wage.

If an employer chooses to pay employees minimum wage, the employer must pay those employees in accordance with the minimum wage law, either federal or state, that results in the employees being paid the higher wage. In most instances in New Mexico, the New Mexico minimum wage will apply as it generally guarantees a higher wage rate for employees than federal law."

New York: "New York's current minimum wage except for fast food workers is as follows:

- New York City – Large Employers (11 or more employees): $11.00
- New York City – Small Employers (10 or fewer employees): $10.50
- Remainder of downstate (Nassau, Suffolk, and Westchester counties): $10.00
- Rest of New York State: $9.70

NY Dept. of Labor – Minimum Wage

The minimum wage rate will increase over the next few years as follows, except for fast food workers:

New York City – Large Employers (11 of more employees)

- December 31, 2017: $13.00
- December 31, 2018: $15.00

New York City – Small Employer (10 or fewer employees)

- December 31, 2017: $12.00

- December 31, 2018: $13.50
- December 31, 2019: $15.00

Remainder of downstate (Nassau, Suffolk, and Westchester counties)

- December 31, 2017: $11.00
- December 31, 2018: $12.00
- December 31, 2019: $13.00
- December 31, 2020: $14.00
- December 31, 2021: $15.00

Rest of New York State

- December 31, 2017: $10.40
- December 31, 2018: $11.10
- December 31, 2019: $11.80
- December 31, 2020: $12.50
- Beginning in 2021, the New York Labor Commission will increase the minimum wage each year for the rest of New York State equal to the percentage change in economic indices, including the consumer price index, until the minimum wage reaches $15.00. The increase will be annouced by October 1 of each year.

Special minimum wage regulations apply to fast food workers, the building service industry and the hospitality industry. Where appropriate, the regulations particular to those industries are discussed in the sections below.

Fast Food Workers Minimum Wage

New York's current minimum wage for fast food workers in New York City is $12.00. For fast food workers in the rest of the state, it is $10.75. NY Fast Food Worker Minimum Wage Fact Sheet

The minimum wage for fast food workers in New York City will increase over the next several years as follows:

- December 31, 2017: $13.50
- December 31, 2018: $15.00

The minimum wage for fast food workers not in New York City will increase over the next several years as follows:

- December 31, 2017: $11.75
- December 31, 2018: $12.75
- December 31, 2019: $13.75
- December 31, 2020: $15.00
- July 1, 2021: $15.00

NY Fast Food Worker Minimum Wage Fact Sheet

A fast food worker for purposes of New York's fast food worker minimum wage is an employee of a fast food establishment. A fast food establishment is defined as a business that:

- Primarily serves food or drinks, including coffee shops, juice bars, donut shops, and ice cream shops;
- offers limited service where customers order and pay before eating, including restaurants with tables but without full table service and places that only provide take-out service; and
- is part of a chain of 30 or more locations, including individually owned establishments associated with a brand that has 30 or more locations nationally.

NY Fast Food Worker Minimum Wage Fact Sheet; NY Admin. Rule 146-3.13"

New York: **(another source)** In NYC minimum salary will increase to $50,700 effective 12/31/17 and to $58,500 effective 12/31/18.

North Carolina: "North Carolina's current minimum wage is $7.25.
North Carolina's Wage and Hour Act exempts many employees covered by the federal Fair Labor Standards Act from its minimum wage, overtime, youth employment, and recording keeping requirements, specifically those employees who work for enterprises engaged in commerce or in the production of goods for commerce as defined in the Fair Labor Standards Act. NC Statute 95-25.14(a)(1) Thus, many employers and employees in North Carolina should refer to the Fair Labor Standards Act and its regulations for purposes of determining minimum wage and overtime rights and responsibilities. There are additional exemptions to North Carolina's minimum wage, overtime, youth employment, and record keeping requirements as set forth below.

North Carolina law requires that its minimum wage be the same as the federal minimum wage set forth in the FLSA if it is higher than the rate set forth in its Wage and Hour Act. NC Statute 95-25.3(a)"

North Dakota: "North Dakota's current minimum wage is $7.25. ND Code 34-06-22(1(c)
North Dakota employers must also comply with federal minimum wage laws, which currently sets the federal minimum wage at $7.25. See FLSA: Minimum Wage.

If an employer chooses to pay employees minimum wage, the employer must pay those employees in accordance with the minimum wage law, either federal or state, that results in the employees being paid the higher wage."

Ohio: "Ohio's current minimum wage is $8.15. Ohio Minimum Wage Poster – 2016
Ohio's constitution requires an annual review of its minimum wage. The minimum wage must be increased by the percentage the cost of living has changed from the prior September to the September in the year the review is conducted. The cost of living change is based on the consumer price index or its successor index for all urban wage earners and clerical workers for all items as calculated by the federal government rounded to the nearest five cents. Any change to the minimum wage takes effect on January 1 of the following year. Ohio Constitution Art. II, Section 34a

Employers who earn less than $297,000 in annual gross receipts need only pay employees the minimum wage set forth in the Fair Labor Standards Act, which is currently $7.25. The amount of gross annual receipts necessary for this exception

increase each year based on the consumer price index as set forth above for Ohio's minimum wage. Ohio Constitution Art. II, Section 34a"

Oklahoma: "Oklahoma's current minimum wage is $7.25.
Oklahoma sets its minimum wage to be the same as the federal minimum wage. Thus, if the federal minimum wage changes, Oklahoma's will change to that same rate as well. OK Statute 40-197.2

Oklahoma's minimum wage act does not apply to employers who have fewer than 10 employees or less than $100,000 of business in a year. It also exempts employers and employees from it minimum wage requirements if they are subject to the Fair Labor Standards Act, so long as they pay employees the minimum wage. OK Statute 40-197.4 Additional exemptions are discussed below."

Oregon: "On March 2, 2016, Gov. Brown signed a law providing for increases to Oregon's minimum wage. The minimum wage increases begin on July 1, 2016, and provide for different minimum wages depending on the area in which a business operates within the state. The increases and geographic distinctions are as follows:
– Employer located within an urban growth boundary of certain metropolitan service districts

- July 1, 2016 – $9.75
- July 1, 2017 – $11.25
- July 1, 2018 – $12.00
- July 1, 2019 – $12.50
- July 1, 2020 – $13.25
- July 1, 2021 – $14.00
- July 1, 2022 – $14.75

– Employers located in certain nonurban counties

- July 1, 2016 – $9.50
- July 1, 2017 – $10.00
- July 1, 2018 – $10.50
- July 1, 2019 – $11.00
- July 1, 2020 – $11.50
- July 1, 2021 – $12.00
- July 1, 2022 – $12.50

Counties that fall within the definition of a "nonurban county" include:

- Baker
- Coos
- Crook
- Curry
- Douglas
- Gilliam
- Grant
- Harney
- Jefferson
- Klamath

- Lake
- Malheur
- Marrow
- Sherman
- Umatilla
- Union
- Wallowa
- Wheeler

– *Employers in any other geographic area*

- July 1, 2016 – $9.75
- July 1, 2017 – $10.25
- July 1, 2018 – $10.75
- July 1, 2019 – $11.25
- July 1, 2020 – $12.00
- July 1, 2021 – $12.75
- July 1, 2022 – $13.50

Beginning in 2023, the minimum wage will increase consistent with an increase in the U.S. City Average Consumer Price Index for All Urban Consumers.

Oregon Senate Bill 1532 – 2016 (Signed by the Governor on March 2, 2016)

Oregon employers must also comply with federal minimum wage laws, which currently sets the federal minimum wage at $7.25. See FLSA: Minimum Wage.

If an employer chooses to pay employees minimum wage, the employer must pay those employees in accordance with the minimum wage law, either federal or state, that results in the employees being paid the higher wage. In most instances in Oregon, the Oregon minimum wage will apply as it generally guarantees a higher wage rate for employees than federal law."

Pennsylvania: "Pennsylvania's current minimum wage is $7.25.
Pennsylvania sets its minimum wage to be the same as the federal minimum wage. Thus, if the federal minimum wage changes, Pennsylvania's will change to that same rate as well.

PA Statute 43-333.104."

Rhode Island: "Rhode Island's current minimum wage is $9.60. RI Dept. of Labor and Training – Minimum Wage
Rhode Island employers must also comply with federal minimum wage laws, which currently sets the federal minimum wage at $7.25. See FLSA: Minimum Wage.

If an employer chooses to pay employees minimum wage, the employer must pay those employees in accordance with the minimum wage law, either federal or state, that results in the employees being paid the higher wage. In most instances in Rhode Island, the Rhode Island minimum wage will apply as it generally guarantees a higher wage rate for employees than federal law."

South Carolina: "South Carolina has not established a state minimum wage rate. Because most employers and employees in South Carolina are subject to the federal Fair Labor Standards Act, the minimum wage set forth in that law would typically apply. Currently, the federal minimum wage is $7.25."

South Dakota: "South Dakota's current minimum wage is $8.65.
Each year, South Dakota increases its minimum wage by any percentage increase in the cost of living. The change in the minimum wage is announced by October 15 each year. SD Department of Labor and Regulation: Minimum Wage

South Dakota employers must also comply with federal minimum wage laws, which currently sets the federal minimum wage at $7.25. See FLSA: Minimum Wage.

If an employer chooses to pay employees minimum wage, the employer must pay those employees in accordance with the minimum wage law, either federal or state, that results in the employees being paid the higher wage."

Tennessee: "Tennessee has not established a state minimum wage rate. Because most employers and employees in Tennessee are subject to the federal Fair Labor Standards Act, the minimum wage set forth in that law would typically apply. Currently, the federal minimum wage is $7.25."

Texas: "Texas's current minimum wage is $7.25.
Texas has adopted, by statute, the federal minimum wage rate set forth in the Fair Labor Standards Act. TX Labor Code 62.051

Texas's minimum wage law does not apply to employees covered by the Fair Labor Standards Act; thus, for most employers and employees, minimum wage obligations are governed by the Fair Labor Standards Act. TX Labor Code 62.151"

Utah: "Utah's current minimum wage is $7.25.
Utah minimum wage laws give authority to the Utah Labor Commission to set the minimum wage; however, the Labor Commission may not set the minimum wage to be higher than the federal minimum wage set forth in the Fair Labor Standards Act. When reviewing the minimum wage, the commission:

- may review it at any time;
- shall review it at least every three years;
- shall review it whenever the federal minimum wage changes.

UT Code 34-40-103; UT Admin. Rules 610-1-3.

Utah minimum wage laws exempt all employees entitled to minimum wage under the Fair Labor Standards Act; thus, because most employees in Utah are subject to the Fair Labor Standards Act, the provisions of Utah's minimum wage law do not apply to most employee working in Utah. UT Statute 34-40-104(1)(a)"

Vermont: "Vermont's current minimum wage is $10.00.

Vermont's minimum wage will increase to $10.50 on January 1, 2018. VT Statute 21-384(a)

For the year beginning January 1, 2019 and every year thereafter, Vermont minimum wage laws will require an annual review of its minimum wage. The minimum wage must be increased by the smaller of the following:

- 5 percent; or
- the percentage increase of the Consumer Price Index, CPI-U, U.S. city average, not seasonally adjusted, or successor index, as calculated by the U.S. Department of Labor or successor agency for the 12 months preceding the previous September 1.

The minimum wage may not be decreased and must be rounded to the nearest $0.01. Any change to the minimum wage takes effect on January 1 of the following year. VT Statute 21-384(a)

Vermont employers must also comply with federal minimum wage laws, which currently sets the federal minimum wage at $7.25. See FLSA: Minimum Wage.

If an employer chooses to pay employees minimum wage, the employer must pay those employees in accordance with the minimum wage law, either federal or state, that results in the employees being paid the higher wage. In most instances in Vermont, the Vermont minimum wage will apply as it generally guarantees a higher wage rate for employees than federal law."

Virginia: "Virginia's current minimum wage is $7.25.
Virginia has adopted the federal minimum wage set forth by the Fair Labor Standards Act as its minimum wage. VA Statute 40.1-28.10. Virginia minimum wage laws exempt all employees entitled to minimum wage under the Fair Labor Standards Act; thus, because most employees in Virginia are subject to the Fair Labor Standards Act, the provisions of Virginia's minimum wage law do not apply to most employee working in Virginia. VA Statute 40.1-28.9(B)(12). Additionally, Virginia minimum wage laws do not apply to employers with fewer than four persons employed at any one time; provided that the employer's spouse, children and parents are not be counted in determining the number of persons employed. VA Statute 40.1-28.9(B)(15)."

Washington: "Washington's current minimum wage is $11.00. WA Dept. of Labor and Industries: Minimum Wage
Washington's minimum wage will increase in coming years as follows:

- January 1, 2018: $11.50
- January 1, 2019: $12.00

Beginning January 1, 2021, Washington minimum wage laws require an annual review of its minimum wage. The review must be completed by September 30 of each year. The minimum wage must be increased by the percentage the cost of living has changed from the prior September 1 to the September 1 in the year the review is conducted. The cost of living change is based on the consumer price index for urban wage earners and clerical workers, CPI-W, or a successor index, as calculated by the U.S. Department of

Labor. Any change to the minimum wage takes effect on January 1 of the following year. WA Code 49.46.020(b); WA Dept. of Labor: Minimum Wage.

Washington employers must also comply with federal minimum wage laws, which currently sets the federal minimum wage at $7.25. See FLSA: Minimum Wage.

If an employer chooses to pay employees minimum wage, the employer must pay those employees in accordance with the minimum wage law, either federal or state, that results in the employees being paid the higher wage. In most instances in Washington, the Washington minimum wage will apply as it generally guarantees a higher wage rate for employees than federal law."

West Virginia: "West Virginia's current minimum wage is $8.75.
If the federal minimum wage is greater than the West Virginia minimum wage, employers must pay the federal minimum wage. WV Code 21-5C-2(a).

West Virginia minimum wage laws do not apply to employers with fewer than six persons employed during a workweek in any one separate, distinct, and permanent location or business establishment. WV Code 21-5C1(e)."

Wisconsin: "Wisconsin's current minimum wage is $7.25. WI Statute 104.02-04; WI Admin. Code 272.03
Wisconsin employers must also comply with federal minimum wage laws, which currently sets the federal minimum wage at $7.25. See FLSA: Minimum Wage.

If an employer chooses to pay employees minimum wage, the employer must pay those employees in accordance with the minimum wage law, either federal or state, that results in the employees being paid the higher wage."

Wyoming: "Wyoming's current minimum wage is $5.15. WY Statute 27-4-202(a).
Wyoming employers must also comply with federal minimum wage laws, which currently sets the federal minimum wage at $7.25. See FLSA: Minimum Wage.

If an employer chooses to pay employees minimum wage, the employer must pay those employees in accordance with the minimum wage law, either federal or state, that results in the employees being paid the higher wage. In most instances in Wyoming, the federal minimum wage will apply as it generally guarantees a higher wage rate for employees than state law."
