# Supplementary material for: Monitoring the compliance of the academic enterprise with the Fair Labor Standards Act
Source: F1000Res. 2017 Sep 28;5:2690. Originally published 2016 Nov 17. [Version 2] doi: 10.12688/f1000research.10086.2 (PMC5130071; doi:10.12688/f1000research.10086.2)
Supplement: Peer review history

# Peer review: Referee response for version 2

**DOI:** 10.5256/f1000research.13681.r26512

**Published:** 2017-10-02

**Authors:** Christopher L. Pickett

**Affiliations:** 1 Rescuing Biomedical Research, Washington, DC, USA

Bankston and McDowell have made considerable modifications, and they have completely addressed my concerns. They also added much more information than was requested, and the resulting document is insightful and thorough. This is an important and impressive amount of work that highlights the inequities in postdoc pay in the biomedical research enterprise.

I have read this submission. I believe that I have an appropriate level of expertise to confirm that it is of an acceptable scientific standard.

# Peer review: Referee response for version 2

**DOI:** 10.5256/f1000research.13681.r26514

**Published:** 2017-10-02

**Authors:** Nathan L. Vanderford

**Affiliations:** 1 Markey Cancer Center, Department of Toxicology and Cancer Biology, College of Medicine, University of Kentucky, Lexington, KY, USA

The revised manuscript has been improved in several ways and now also contains information/data regarding the planned institutional changes in relation to the injunction against the FLSA updates. The manuscript provides an important analysis and it raises many additional questions. In relation to the latter comment, as I stated in my initial review, I believe it would be useful for the authors to clearly discuss policy recommendations related to the pay and employment status of postdocs. For example, the issue of some institutions recognizing postdocs as employees and other institutions not doing so is an issue that may require greater policy clarification at the federal level. What would the authors recommend in relation to this?

 Minor comment:

 The authors should check for spelling and typo errors. For example, “possible” is misspelled in the second paragraph of the conclusion.

I have read this submission. I believe that I have an appropriate level of expertise to confirm that it is of an acceptable scientific standard.

# Peer review: Referee response for version 1

**DOI:** 10.5256/f1000research.10867.r18035

**Published:** 2016-11-28

**Authors:** Paula E. Stephan

**Affiliations:** 1 Department of Economics, Andrew Young School of Policy Studies, Georgia State University, Atlanta, GA, 30303, USA

The Bankston & McDowell article is highly informative on two counts. First, it highlights the extreme difficulty encountered in collecting information regarding postdocs. The Boston data are but one case in point.  Second, it contributes information concerning the percent of postdocs which, at the time Bankston & McDowell collected their data, were likely to be affected by the FLSA ruling. This constitutes a significant contribution to our understanding of how the law may affect actual salaries.

 For my taste, however, the article could go a bit further. First, the authors present no information regarding who will pay for the increase on campuses that intend to raise salaries sufficiently so that postdocs will be exempt. The authors speculate that the funds will come out of grants. And in the long run, they are probably right. But, in the short run the evidence is that universities and departments will pick up some of the increases. (See Council on Governmental Relations 2016 Survey). Second, they do not speculate on how the increase will likely affect the number of postdocs employed. To the extent the demand for postdocs is sensitive to salary, one would predict that in the long run the regulation will lead to a decrease in the number of postdocs working at universities. 

 The authors also imply that it is not clear as to whether the regulation applies to postdocs on fellowships. It is my understanding that a decision by the Department of Labor means that those on fellowships are to be treated the same as are postdocs supported in other ways.

 The authors also make no effort to explain why their results seem so at odds with the results found by the Council on Governmental Relations Survey, which suggests that something like 75% of campuses surveyed were choosing to raise salaries so that postdocs would be exempt from overtime pay.

 Finally, there is the issue that the authors could not have foreseen that a Federal Judge would issue an injunction November 22, 2015 which preserves the status quo. While the injunction is temporary, many believe that the judge’s language “indicated he was likely to strike down the regulation.” http://www.nytimes.com/2016/11/22/business/obama-rule-to-expand-overtime-eligibility-is-suspended-by-judge.html.

 To the extent the implications of the regulation for postdoc pay were difficult to understand before this ruling, they are even more so today. Assuming the rule is found to be invalid, campuses will find themselves in the awkward position of having to decide if they will take back a promised increase. Or, take back an increase that has already taken effect.

I have read this submission. I believe that I have an appropriate level of expertise to confirm that it is of an acceptable scientific standard.

# Peer review: Referee response for version 1

**DOI:** 10.5256/f1000research.10867.r17778

**Published:** 2016-11-22

**Authors:** Christopher L. Pickett

**Affiliations:** 1 Rescuing Biomedical Research, Washington, DC, USA

The research article “Monitoring the compliance of the academic enterprise with the Fair Labor Standards Act” by Bankston & McDowell is a review of academic compliance with changes to the FLSA overtime rules as of Nov. 1. The authors give a good primer on the history of the FLSA overtime rule change and how it is expected to affect academic research starting Dec. 1. They then discuss their extensive outreach to institutions with postdocs, and their efforts to determine how the institutions are adjusting to the new overtime rule with regard to postdoc salaries.

 This paper and the results of the authors’ outreach are important for the postdoc and larger academic communities to understand how institutions are dealing with the increase to postdoc pay.

 Major concerns:

- The authors should devote some space to a discussion of international postdocs and how they are affected by the FLSA overtime rule change. Anecdotally, international postdocs may not be paid on the same scale as American postdocs, even at institutions with well-defined salary minima. Since the nation of origin does not affect whether someone is FLSA exempt, just how widespread the disparities in U.S. versus international postdoc pay are could have significant repercussions in the relative populations of each in the enterprise moving forward.
- The authors should take the time to explain exactly what is going on in Boston. There seems to be an assumption that the reader will know all of the institutions that are classified under “Harvard” or “MIT”. The assumption comes across in phrases such as institutions “…cannot be more than a small proportion…” As someone who has never done research in Boston, it is not clear to me why this phrase must be true. The authors should take the time to indicate, both in the text and Table 1, which institutions are part of “Harvard” for example, which have released decisions about postdoc pay, and why this may be confusing for postdocs employed there.
- The conclusions section opens with a meandering and confusing sentence and the first section is about the difficulties of data collection. I agree these are real concerns, but the authors should state their overall and most important conclusions at the beginning of this section as a way to frame the rest of the discussion.

 

 Minor concerns

- Background
  - The sentence beginning, “The other key change is indexing…” is not clear. Recommend breaking the sentence into two with the second sentence starting something like, “This means the OT threshold will be $51,168 in 2020.”
  - It would be helpful to know the current status of H.R. 6094. Was it vetoed or does it await action?
  - On page 4, in the paragraph about the test that must be passed to be eligible for overtime pay, the authors should consider a short example at the end of the paragraph. For example, “A first year postdoc in 2015 earning a salary of $XX,XXX would pass the salary basis test, would fail the salary level test and pass the standard duties test. Hence the focus on the overtime pay threshold.”
  - In the paragraph starting “The minimum salary should therefore…”: for consistency, recommend the authors indicate years experience as they do in Fig. 1 (Year 0, 1, etc.) as opposed to calling them “new postdocs”.
  - The two problems with the assumption of the NRSA minimum as the actual minimum pay for postdocs, as I understand them are, (1) transparent salary information does not exist and (2) institutional salary minima are unknown or unenforced. These seem very similar problems to me and I don’t understand the distinction the authors are trying to make. I recommend the authors draw a starker division between the two problems they see or discuss them as one.
- Due to the date-sensitive nature of the information, I recommend the authors add the date to the titles of all figures and tables.
- In the discussion, the comparison between medical residents and postdocs:
  - The authors should explicitly state why, after each condition they site, why postdocs would be more likely to report violations. For example, “…debts in the academic path, meaning postdocs are significantly less dependent on academic employers and may be more inclined to report violations.” “…postdocs end up leaving academia meaning postdocs are less likely to be concerned about how academic employers will view their willingness to report violations.”
  - “…compared to the relatively lower (undergraduate) student debts…” It is not clear why the authors specify these as undergraduate debts. Graduate students can also secure student loans. I recommend removing the parenthetical.

I have read this submission. I believe that I have an appropriate level of expertise to confirm that it is of an acceptable scientific standard, however I have significant reservations, as outlined above.

# Peer review: Referee response for version 1

**DOI:** 10.5256/f1000research.10867.r17777

**Published:** 2016-11-18

**Authors:** Nathan L. Vanderford

**Affiliations:** 1 Markey Cancer Center, Department of Toxicology and Cancer Biology, College of Medicine, University of Kentucky, Lexington, KY, USA

Bankston and McDowell provide a comprehensive and well-written report on the status and impact of the Fair Labor Standards Act (FLSA) on the US postdoc population. This article is very timely given the “go-live” date for implementing this Act is less than a month away and given that there has been great consternation among academic institutions in terms of how the mandates will be implemented and what impacts it will have on institutions, postdocs, and science in general.

 While acceptable for publication now, there are minor changes that could improve the article.

1. The non-yes (i.e, the “X” and “-“) annotations in dataset 1 should be defined.
2. The y-axis of Figure 1 should be labeled.
3. At the top of page 7, a deeper analysis comparing salary by region, public vs. private institutions, etc. is mentioned as a future direction. This is a critical aspect of understanding how the FLSA will differentially impact institutions and postdocs across the country. I believe it is a very important point that, for example, the cost of living in the middle of the country is not comparable to the cost of living on the east and west coast, which begs the question of whether such differences should be considered within the FLSA. Will this analysis be added to this current article as more data is collected or will this form the basis of another article? It is my opinion that this analysis would greatly strengthen and compliment the current article’s argument and impact.
4. It may be interesting (and impactful) to add a current snapshot of what postdocs think of the FLSA implementation at their institutions. At my institution, postdocs have been very concerned about the short- and long-term impacts on their current positions and how any potential changes (eg, losing their position) may impact their career progression. It would be interesting to hear from postdocs representing institutions across the country (not just on the coasts).
5. At the bottom of page 8 the authors begin to question how the FLSA will impact the biomedical enterprise. It would be more powerful if the authors were more definitive and concrete with their own opinions as to what impact FLSA will have on science. Some have argued that the FLSA will shrink the postdoc pool thus shrink the pipeline of future researchers thus set back the momentum of new science discoveries. The article could be more impactful if the authors wrote specific speculations as to the threats and opportunities that the FLSA will have on the biomedical enterprise.
6. Similar to point #4, could the authors provide some specific recommendations to institutions, postdocs, the government, etc.?
7. What implications will/could a new political party and leader have on FLSA?

 

 In summary, this is an important article that is acceptable for publication now although there are several areas that could be improved upon as noted above. As such, I look forward to reviewing any revised version of the article.

I have read this submission. I believe that I have an appropriate level of expertise to confirm that it is of an acceptable scientific standard.
